# Quality of YouTube TM videos on dental implants

**DOI:** 10.4317/medoral.22447

**Published:** 2018-06-21

**Authors:** Ashraf Abukaraky, Ahmad A. Hamdan, Mohammed-Noor Ameera, Mahmoud Nasief, Yazan Hassona

**Affiliations:** 1 Msc, Department of oral and Maxillofacial Surgery, Oral Medicine and Periodontology, School of Dentistry, The University of Jordan, Amman; 2 PhD, Department of oral and Maxillofacial Surgery, Oral Medicine and Periodontology, School of Dentistry, The University of Jordan, Amman; 3 DDS, Department of oral and Maxillofacial Surgery, Oral Medicine and Periodontology, School of Dentistry, The University of Jordan, Amman

## Abstract

**Background:**

Patients search YouTube for health-care information.

**Purpose:**

To examine what YouTube offers patients seeking information on dental implants, and to evaluate the quality of provided information.

**Material and Methods:**

A systematic search of YouTube for videos containing information on dental implants was performed using the key words “Dental implant” and “Tooth replacement”. Videos were examined by two senior Oral and Maxillofacial Surgery residents who were trained and calibrated to perform the search. Initial assessment was performed to exclude non- English language videos, duplicate videos, conference lectures, and irrelevant videos. Included videos were analyzed with regard to demographics and content’s usefulness. “Information for patients” available from the American Academy of Implant Dentistry, European Association of Osseointegration, and British Society of Restorative Dentistry were used for benchmarking.

**Results:**

A total of 117 videos were analyzed. The most commonly discussed topics were related to procedures involved in dental implantology (76.1%, n=89), and to the indications for dental implants (58.1%, n=78). The mean usefulness score of videos was poor (6.02 ±4.7 [range 0-21]), and misleading content was common (30.1% of videos); mainly in topics related to prognosis and maintenance of dental implants. Most videos (83.1%, n=97) failed to mention the source of information presented in the video or where to find more about dental implants.

**Conclusions:**

Information about dental implants on YouTube is limited in quality and quantity. YouTube videos can have a potentially important role in modulating patients’ attitude and treatment decision regarding dental implants.

** Key words:**YouTube, Social Media, Dental Implant, Rehabilitation, Patient information, E-health.

## Introduction

Dental implants are increasingly used for oral rehabilitation of partially dentate or edentulous patients (1). Osseointegrated dental implants offer patients a more satisfactory option to replace missing teeth because they preserve the structure of adjacent teeth, and provide a better comfort, aesthetic outcome, function, and stability with a highly predictable 10-year survival rate of approximately 90% (2). Patient education is an essential element for the success of any treatment plan, and is particularly important in dental implantology.

The success of dental implants depends on thorough assessment of the patient’s conditions and careful evaluation of indications and contra-indications (3). Previous studies reported an inadequate knowledge of dental patients with regards to different aspects of implant dentistry (4-6). In fact, information on dental implants is widely available in the public domain from different resources including the industry, dental practitioners, heath insurance companies, academic institutions, and professional organizations. Interestingly, more patients are browsing the internet to seek information on various health related issues including dental implants. A recent study has demonstrated that the exposure of the public to such information may form a basis for their perceptions of dental implants and affect their intentions to consider dental implants as a treatment option when the need arises (7). In addition, such information may influence the communications of patients with dentists, and their decision-making between different implant treatment options (7). Interestingly, Ho et al evaluated the social media testimonials of dental implants patients and concluded that the potential of these testimonials to provide educational value is limited, and many important parameters of implant therapy are overlooked, whilst information is often potentially misleading (8).

YouTube TM is a very popular web resource where patients can search for information on dental implants. Importantly, YouTube TM videos are not subjected to peer-reviewing; therefore, patients browsing YouTube TM for health care information might encounter inaccurate and potentially misleading content.

Several studies evaluated YouTube TM content in various medical aspects, but only few studies evaluated oral health related videos on YouTube TM (9-15). Our study aimed at analyzing the content of YouTube TM Videos regarding the type and quality of information they contain about dental implantology.

## Material and Methods

We have recently described a systematic approach for searching YouTube TM content (12); a similar approach was adopted in the present study. In brief, two search terms “Dental implant” and “Tooth replacement” were used to conduct a YouTube TM search at 10:00 am GMT on the 22 of March 2017 using the default settings of YouTube TM with “relevance” sorting, and with applying no search filters. The two search terms were adopted in the present study because they were the most commonly used search terms for dental implantology according to ‘Google Trends’ application. The first 160 videos appearing for each search term (total of 320 videos) were viewed and analyzed; most studies utilizing YouTube TM as a search engine have used 60-200 videos (16), and the majority of YouTube TM users scan only the first 30 videos (17). Two senior residents (M.N.A & M. N) in Oral and Maxillofacial Surgery performed initial screening of videos to exclude non- English language videos, duplicate videos, conference lectures, and irrelevant videos. The two researchers were trained and calibrated to perform the search, and Kohen’s kappa coefficient was used to examine inter-examiner agreement.

The remaining videos were analyzed for the quality and usefulness of information they contain.

“Information for patients” available from the American Academy of Implant Dentistry, European Association of Osseointegration, and British Society of Restorative Dentistry were used for benchmarking. We assessed videos for the presence of content in ten non-mutually exclusive domains of implant-related information ( Table 1). The quality of data contained in each domain was assessed using a 4-point score ( Table 2), and each video was given a total score that ranged from 0 to 30. Score 0 indicated that the video contained no information related to any of the 10 domains assessed, or it contained misleading information on all assessed domains; score 30 indicated that the video contained comprehensive and scientifically valid information on each domain assessed. Disagreements between researchers regarding scoring of a particular video were solved by discussion of the literature until a consensus was reached.

Viewers’ interaction was calculated using the formula we previously described ((number of likes-number of dislikes/total number of views) *100%) and the viewing rate ((number of views/number of days since upload) *100%). Descriptive statistics were generated using GraphPad Prism 6.0 (GraphPad Software Inc, California USA), and Pearson’s test was used to examine correlation between variables. No ethical approval was deemed necessary for this study.

**Table 1:**
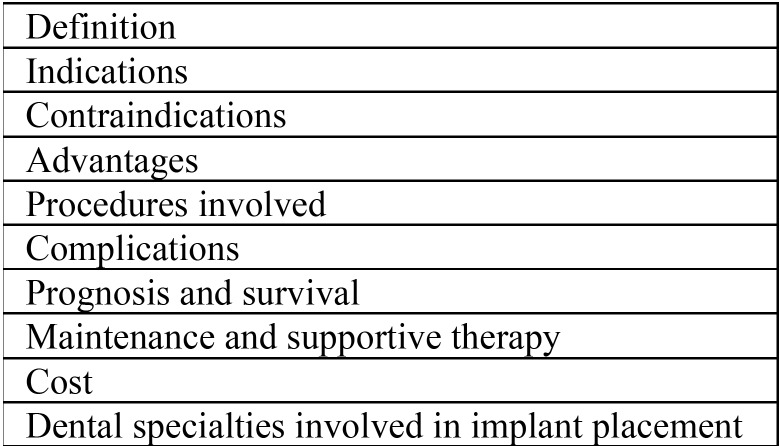
Topics domains evaluated in YouTube TM videos about dental implants.

**Table 2:**
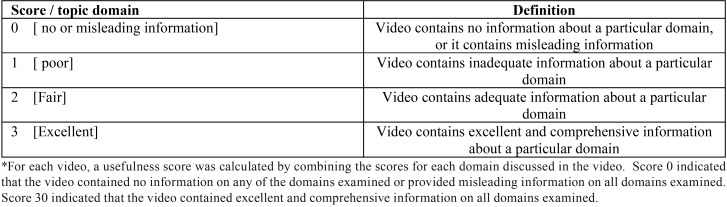
YouTube video usefulness scoring.

## Results

**Figure 1:**
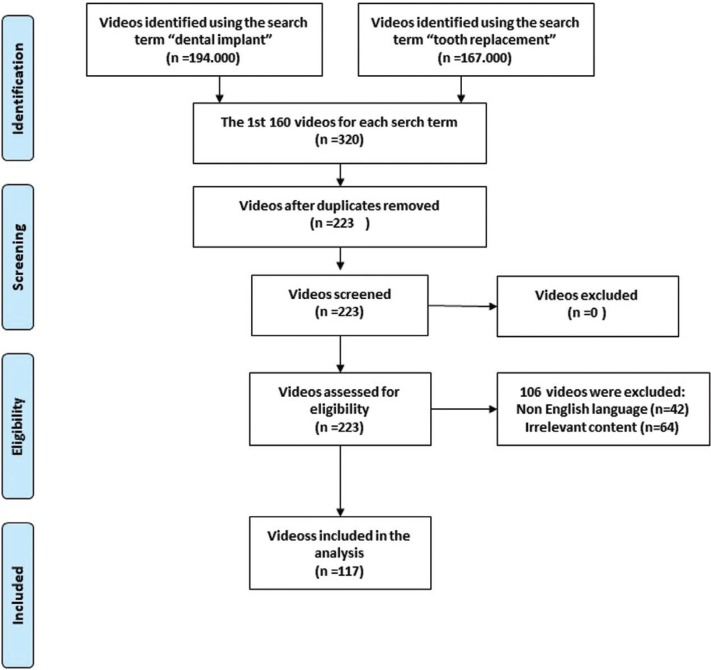
Search strategy.

The search term “dental implant” yielded a total of (194.000) videos, and the search term “Tooth replacement” yielded a total of (167.000) videos. Out of the 320 videos initially screened, 203 videos were excluded for reasons shown in Figure 1. The remaining 117 videos were subjected to further analyses.

More than half of the videos (46.9%, n=76) were uploaded by health care professionals; 15.4% (n=18) by individual users, 13.7% (n=16) by health companies, 4.3% (n=5) by T.V channels, and 0.9% (n=1) by academic institutions. Most videos (87.1%, n=102) were uploaded by users from USA and Canada. The mean viewing length of YouTube TM videos on dental implants was 6.16 minutes (range 1.14-55.1minutes; median= 7.9). The total number of views for dental implant related videos was 6915955 views (range 40-1051024 views); each video was viewed on an average of 3.5 views/day (range: 0.62-42.3views/day; median=4.1).

**Figure 2:**
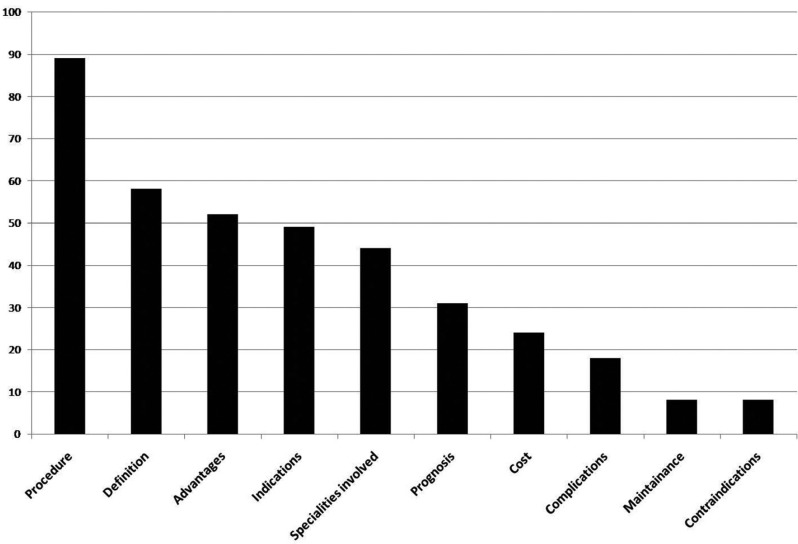
YouTube TM topics related to dental implants.

YouTube TM videos contained variable information on dental implants (Fig. 2); a YouTube TM video on dental implants discussed an average of 3.6 domains. The most commonly discussed topics were related to procedures involved in dental implantology (76.1%, n=89), and to the indications for dental implants (58.1%, n=78). Few videos (6.8%, no=8) contained information on the importance of maintenance of dental implants, (6.8%, n=8), described contra-indications (metal allergy, uncontrolled systemic disease, and advanced periodontitis), and (15.4%, n=18), mentioned potential complications (implant failure, infection, pain, and damage to nerves). Some YouTube TM videos (20.5%, n=24) contained information on the cost of dental implants; prices quoted ranged almost 10-fold, from 750$/implant for implants placed by students in dental schools to 6500$/implant in private practices. Videos uploaded by individual users advised patients to beware of hidden costs such as those of sedation, hotel accommodation, travel cost, additional procedures such as bone or soft tissue grafts. Videos uploaded by health care professionals often contained “price offers” such as discounts or payment over 24 months with 0% interest for multiple implants. YouTube TM videos (37.5%, n=44) contained information on where to go for dental implant placement; most videos recommended periodontists (n=28), oral surgeons (n=21), or trained general dental practitioners (n=19).

Misleading information existed in 30.1% (n=35) of videos; mainly in topics related to prognosis and maintenance of dental implants. Most videos (83.1%, n=97) failed to mention the source of information presented in the video or where to find more about dental implants.

The mean interaction index score was 0.25%±0.38 (range from 0 to 3.4%; median= 0.16), and the mean usefulness score of videos was 6.02 ±4.7 (range from 0-21; median= 7.08). Pearson’s correlation analysis showed no significant correlation between the usefulness score and source of upload (r=0.08, *p*>0.05), viewing rate (r=0.09, *p*>0.05), interaction index score (r= 0.07, *p*>0.05), and video length (r=0.14, *p*>0.05).

The overall inter-observer agreement calculated as kappa score was 0.76. Intra-observer reliability, calculated for each researcher as kappa score, was 0.93 for MNA and 0.84 for M.N.

## Discussion

YouTube TM contains information on various topics related to dental implants; most videos contained description of the surgical and prosthetic procedures in implant dentistry. Importantly very few videos contained advice on maintenance of dental implants including the need for frequent recall visits and oral hygiene, yet several studies have demonstrated that inadequate plaque control and lack of supportive therapy is a major risk indicator for the development of peri-implantitis, and ultimately implant failure (18-20).

Risk factors for dental implants were barely discussed in YouTube TM videos; our study revealed that only a minority of videos contained information on contraindications/precautions for dental implants. Interestingly, the literature contains very few evidence- based data on the contraindications for dental implants, yet generally it is agreed that a poor outcome might be expected in patients with poor oral hygiene, active periodontal disease, heavy smoking, uncontrolled systemic diseases, cancer chemotherapy, active metabolic bone disease, increasing age, immunosuppression, cardiovascular condition, and hepatitis (21-23).

Dental implants however, have a high success rate. In our search only few videos contained information on prognosis and outcome of dental implants placement; and some of these videos contained misleading information such as incorrect figures relating to success rate and incidence of implant failure. Such inaccurate data might discourage patients from considering dental implants for tooth replacement. A recent study by Wang et al reported that patients tend to over-estimate the function and longevity of dental implants (7). The availability of scientifically valid data on the prognosis of dental implants is therefore important.

Dental implants are relatively expensive, and in most countries they are not included in dental health insurance. Patients often search the internet to find an estimated cost for dental implant therapy. Our study showed that only few videos contained information about an approximate cost for a dental implant. The quoted prices ranged from 750$ to 6500$. The cost of dental implants varies from one country to another and many patients are willing to travel to get dental implant treatment at a more affordable cost (24).

The growing popularity of dental implants has led to an increase in the number of dentists trained in implant dentistry. YouTube TM videos encouraged patients to look for a trained professional (periodontists, oral surgeon, qualified GDP) for dental implant placement.

The search strategy adopted in the present study was extensive, and videos were objectively evaluated for usefulness using a novel scoring system that showed a satisfactory inter-observer and intra-observer agreement. The present study however possesses several limitations. First, study results may change according to the key words used in the search. In the present study we performed two independent searches using the key words “dental implant” and “tooth replacement” which are the most likely keyword a lay person would use when searching YouTube TM on this topic. Some patients however might use other search terms and might get different results. Second, content of YouTube TM is highly dynamic, and videos are added and deleted all the time. Results therefore might vary according to the search date and time. Like similar studies, our study suffers from the ‘snap shot’ approach to data collection. Future studies are therefore encouraged to adopt a longitudinal or field-based approach to study the usefulness of YouTube TM as a source for patient education about dental implants. Third, we analyzed English language videos only. USA and European countries, particularly the UK, have the highest internet penetration rates; it is not surprising therefore to know that the majority of videos stored on YouTube TM are English language videos (25).

## Conclusions

YouTube TM videos on dental implants were limited in the quantity and quality of data they contain. Usefulness scores ranged from 0 -21 with a mean of 6.02 out of 30 indicating generally low scores. This correlates with studies evaluating YouTube TM videos regarding other healthcare issues where the content was found to be generally poor.
